# Biomarkers of Inflammation and Association with Cardiovascular Magnetic Resonance Imaging for Risk Stratification and Outcome in Patients with Severe Aortic Stenosis

**DOI:** 10.3390/jcm14072512

**Published:** 2025-04-07

**Authors:** Matthias Koschutnik, Christina Brunner, Christian Nitsche, Carolina Donà, Varius Dannenberg, Kseniya Halavina, Sophia Koschatko, Charlotte Jantsch, Katharina Mascherbauer, Christina Kronberger, Michael Poledniczek, Caglayan Demirel, Dietrich Beitzke, Christian Loewe, Christian Hengstenberg, Andreas A. Kammerlander, Philipp E. Bartko

**Affiliations:** 1 Department of Internal Medicine II, Division of Cardiology, Medical University of Vienna, 1090 Vienna, Austria; matthias.koschutnik@meduniwien.ac.at (M.K.); christian.hengstenberg@meduniwien.ac.at (C.H.); philippemanuel.bartko@meduniwien.ac.at (P.E.B.); 2 Department of Biomedical Imaging and Image-Guided Therapy, Division of Cardiovascular and Interventional Radiology, Medical University of Vienna, 1090 Vienna, Austria

**Keywords:** aortic stenosis, leukocyte indices, CMR, systemic inflammation, outcome

## Abstract

**Background**: Inflammatory indices have been proposed as simple and routinely obtainable markers of systemic inflammation in cardiac disease. This study investigated whether the neutrophil-to-lymphocyte ratio (NLR), the monocyte-to-lymphocyte ratio (MLR), and the pan-immune inflammation value (PIV) serve as biomarkers for risk stratification and outcomes measures in patients with severe aortic stenosis (AS) following valve replacement (AVR). **Methods**: In this retrospective analysis (January 2017–June 2022), patients with AS underwent pre-procedural cardiovascular magnetic resonance (CMR) imaging and were assigned a treatment strategy by a multidisciplinary Heart Team: (1) transcatheter AVR, (2) surgical AVR, or (3) no valvular intervention. Kaplan–Meier estimates and regression analyses were used to demonstrate associations between the NLR, MLR, and PIV with myocardial fibrosis—assessed by late gadolinium enhancement (LGE) and extracellular volume (ECV) on CMR—and a combined endpoint of heart failure hospitalizations and all-cause mortality. **Results**: A total of 356 patients (median age: 80 years, 50% male) were followed for a median of 40 months, during which 162 (46%) reached the combined endpoint. Linear regression identified C-reactive protein, but not the presence of LGE or elevated ECV, as the only independent predictor of all three inflammatory indices (*p* for all <0.001). After multivariable adjustment for clinical (EuroSCORE II), laboratory (baseline N-terminal prohormone of brain natriuretic peptide [NT-proBNP] and C-reactive protein), and imaging parameters (AV mean pressure gradient, right ventricular ejection fraction, and ECV), the above-the-upper-quartile NLR (adjusted hazard ratio [aHR]: 1.45, 95%-confidence interval [CI]: 1.01–1.92, *p* = 0.042), MLR (aHR: 1.48, 95%-CI: 1.05–2.09, *p* = 0.025), and PIV (aHR: 1.56, 95%-CI: 1.11–2.21, *p* = 0.011) remained significantly associated with adverse outcomes. Following AVR, the median NLR (3.5 to 3.4) and PIV (460 to 376) showed a significant post-procedural decline compared to baseline (*p* ≤ 0.019 for both). **Conclusions**: Inflammatory indices are readily available biomarkers independently associated with adverse outcomes in severe AS following AVR. However, no significant relationship was observed between the NLR, MLR, PIV, and myocardial fibrosis on CMR.

## 1. Introduction

Severe aortic stenosis (AS) represents the most common valvular heart disease in the industrialized world, affecting 3–4% of individuals aged 75 and older [1,2]. The expanding availability of treatment options, including surgical (SAVR) or the less invasive method of transcatheter aortic valve replacement (TAVR), highlights the importance of improved risk stratification optimizing outcomes in this increasingly diverse patient population.

Systemic inflammatory markers have been identified as potential predictors of adverse outcomes across various cardiac and non-cardiac conditions [3,4,5]. Prior studies have demonstrated associations between inflammatory indices, including the neutrophil-to-lymphocyte ratio (NLR), the monocyte-to-lymphocyte ratio (MLR), and the pan-immune inflammation value (PIV), and clinical outcomes in heart failure (HF) [6,7] and coronary artery disease [8,9].

Cardiovascular magnetic resonance (CMR), including late gadolinium enhancement (LGE) and the quantification of extracellular volume (ECV), is well established as non-invasive gold standard for assessing myocardial fibrosis in valvular heart disease [10,11]. While systemic inflammation is believed to contribute to fibrotic changes [12], its relationship with cardiac remodeling in AS is unknown.

At the molecular level, chronic inflammation drives AS progression, facilitating the transition from sclerosis to stenosis [13]. Endothelial injury promotes lipid infiltration, inflammatory cell recruitment, and activation of valve interstitial cells (aVICs), leading to fibrosis and ultimately calcification [14]. Similarly, inflammatory processes both drive and arise from HF, playing a pivotal role in its pathogenesis and prognosis [15]. However, the precise role of inflammatory biomarkers in AS progression remains unclear.

We hypothesized that elevated inflammatory indices are associated with increased myocardial fibrosis on CMR and adversely impact outcomes in patients with severe AS undergoing AVR.

## 2. Materials and Methods

### 2.1. Study Design

This retrospective observational study was conducted within a prospective patient registry at the Medical University of Vienna, Austria, a university-affiliated tertiary care center equipped with a multimodality imaging laboratory and a high-volume cardiac catheterization unit. For this analysis, we included all patients with severe AS referred for multidisciplinary Heart Team evaluation between January 2017 and June 2022 who underwent routine pre-procedural CMR to accurately assess ventricular size, function, and myocardial fibrosis. Treatment strategies included TAVR, SAVR, or conservative management. Patients were excluded if a complete hemogram was unavailable.

The investigation adheres to the principles of the Declaration of Helsinki, and the study protocol received approval from our Institutional Review Board (identifier: EK 2218/2016, amended version 03/2023). Written informed consent was obtained from all participants prior to enrollment.

### 2.2. Study Procedures

Baseline assessment consisted of clinical evaluation with extensive laboratory testing, including assessment of blood chemistry, N-terminal prohormone of brain natriuretic peptide (NT-proBNP) levels, and an automated differential hemogram. Blood was sampled into ethylenediaminetetraacetic acid (EDTA) tubes and subsequently processed at the Department of Laboratory Medicine of the Medical University of Vienna. Differential blood counts were performed using Sysmex XE and XN series hematology analyzers (Sysmex Corporation, Kobe, Japan). The estimated glomerular filtration rate (eGFR) was calculated utilizing the Chronic Kidney Disease Epidemiology Collaboration (CKD-EPI) equation. Baseline evaluations were conducted during the initial patient encounter, either at the outpatient clinic or upon first inpatient admission.

### 2.3. Echocardiography

Comprehensive echocardiographic assessments, including the quantification of valvular heart disease, were conducted by board-certified cardiologists using high-end scanners (e.g., Vivid E95 and Vivid 7, GE Healthcare, Wauwatosa, WI, USA) in accordance with current guidelines and recommendations [16,17]. Post hoc image analysis was performed on an offline clinical workstation utilizing dedicated software (EchoPAC, Version 206, GE Healthcare, Wauwatosa, WI, USA).

### 2.4. Cardiovascular Magnetic Resonance

All CMR studies were conducted using a 1.5 Tesla dedicated cardiac scanner (MAGNETOM Avanto FIT, Siemens Healthineers, Erlangen, Germany), following standardized protocols [18]. This included LGE imaging with gadubutrol (Gadovist, Bayer Vital GmbH, Leverkusen, Germany) for patients with preserved renal function (eGFR > 30 mL/min/1.73 m^2^). At the time of intravenous cannula insertion, blood samples were collected for hematocrit and serum creatinine measurements. The presence of LGE was assessed on short-axis image stacks using a semiautomatic approach, applying a threshold of 5 standard deviations (SDs) above the mean signal intensity of healthy myocardium [19].

T1 mapping was performed using an electrocardiographically triggered modified Look-Locker inversion recovery (MOLLI) sequence with a 5(3)3 prototype (5 acquisition heartbeats followed by 3 recovery heartbeats and an additional 3 acquisition heartbeats) on a mid-cavity short-axis slice and a four-chamber view. This protocol incorporated inline motion correction and calculation of the T1 relaxation curve within a single breath-hold. T1 sequence parameters included an initial inversion time (TI) of 120 ms, TI increment of 80 ms, reconstructed matrix size of 256  ×  218, and acquired matrix size of 256  ×  144 (phase encoding resolution: 66%; phase encoding field of view: 85%). T1 maps were acquired both before and 15 min after contrast agent administration. For post contrast T1 mapping, a 4(1)3(1)2 prototype was used. Regions of interest (ROIs) were defined as left ventricular (LV) myocardium without visually detectable LGE, not detectable by visual assessment and without areas of scar. T1 values for the blood pool were obtained while maintaining sufficient distance from the papillary muscles and endomyocardial border. T1 values from the short-axis and four-chamber views were averaged. ECV was calculated using the following formula [20]:

$$\mathrm{ECV}=\left(1-\mathrm{hemocrit}\right)\times \frac{\left(\frac{1}{T1\;\mathrm{myo}\;\mathrm{post}}\right)-\left(\frac{1}{T1\;\mathrm{myo}\;\mathrm{pre}}\right)}{\left(\frac{1}{T1\;\mathrm{blood}\;\mathrm{post}}\right)-\left(\frac{1}{T1\;\mathrm{blood}\;\mathrm{pre}}\right)}$$

where “T1 myo pre” and “T1 blood pre” represent native T1 times of the myocardium and blood, respectively, while “T1 myo post” and “T1 blood post” represent their respective values 15 min post-contrast administration. In accordance with our institutional standards and based on the current literature, cut-offs of native two-chamber myocardial T1 times of 972 ms and an ECV of 26% were considered normal [21].

Image analysis, including qualitative LGE assessment, was conducted by CN and AAK, both with over seven years of experience. All additional CMR analyses were performed using specialized software (cmr42, Circle Cardiovascular Imaging Inc., Calgary, AB, Canada).

### 2.5. Biomarkers of Inflammation

Inflammatory indices were derived from baseline laboratory assessments. The NLR was calculated by dividing the absolute neutrophil count (G/L) by the absolute lymphocyte count (G/L). Similarly, the MLR was determined by dividing the absolute monocyte count (G/L) by the absolute lymphocyte count (G/L). The PIV was calculated as the product of the neutrophil count, monocyte count, and platelet count, divided by the lymphocyte count (G/L for all values).

### 2.6. Outcome Measures

Patients were prospectively followed after AVR at a dedicated outpatient clinic, with follow-up visits at 3 months, 12 months, and yearly thereafter. The primary outcome measure was a combined endpoint of HF hospitalizations and death. HF hospitalizations were defined as inpatient admission with clinical signs and symptoms of HF, requiring intravenous diuretic treatment. In addition, all-cause mortality was explored as a secondary endpoint. Outcome data were collected through follow-up visits, state-wide electronic hospital records, and direct patient phone calls. Mortality data were obtained from the National Registry of Deaths (Statistics Austria). In an exploratory analysis, we assessed changes in inflammatory indices before and at least one month after AVR to minimize bias associated with the immediate postoperative period. An internal adjudication committee, comprising CD and CH, who were blinded to imaging and procedural data, confirmed all endpoints.

### 2.7. Statistical Analysis

Continuous data are presented as median (interquartile range [IQR]), and categorical variables as counts and percentages, respectively. Comparisons between groups were performed using either Chi-squared or Fisher’s exact tests for categorical variables or Wilcoxon rank-sum tests for continuous variables, as appropriate. Spearman’s and intraclass correlation coefficients were utilized for correlation analyses. Correlations were categorized as “weak” (0.00–0.39), “moderate” (0.40–0.59), and “strong” (0.60–1.00). Predictors of the inflammatory indices were explored using linear regression analyses. Kaplan–Meier curves were plotted, and the Log-rank test was applied to estimate differences between survival curves. Cox regression models were calculated to investigate the association between the NLR, MLR, and PIV, and the combined endpoint of HF hospitalizations and death. All parameters were tested in a univariable model. Parameters with significant predictive value in the univariable Cox regression were included in a multivariable Cox regression model. Wilcoxon signed-rank tests were used to compare inflammatory indices between baseline and follow-up. A two-sided *p*-value < 0.05 was considered statistically significant. All analyses were performed using SPSS 29 (IBM Corporation, Armonk, NY, USA) and STATA 15.1 (StataCorporation, College Station, TX, USA).

## 3. Results

### 3.1. Baseline Characteristics

**Figure 1:**
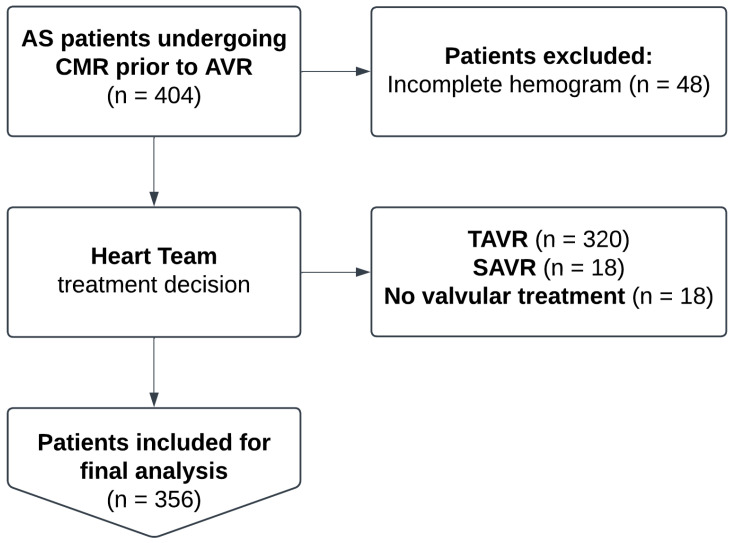
Study workflow. Abbreviations: AS indicates aortic stenosis; CMR, cardiovascular magnetic resonance; AVR, aortic valve replacement; TAVR, transcatheter AVR; SAVR, surgical AVR.

A total of 404 consecutive patients with AS undergoing CMR were screened between January 2017 and June 2022, of whom 356 (88.1%) were included in the final analysis. Further, 48 (11.9%) individuals were excluded due to an incomplete hemogram. Based on the Heart Team’s decision, 320 (89.9%) patients underwent TAVR, 18 (5.1%) SAVR, and 18 (5.1%) received no treatment for valvular heart disease. The details of the study workflow are outlined in Figure 1.

Baseline characteristics of the study population are summarized in Table 1**.** The median age was 80 (IQR: 77–85) years, and 177 (50%) patients were male. Overall, individuals were estimated to be at intermediate to high risk of death, as assessed by EuroSCORE II (4.1%), and presented with elevated median NT-proBNP levels (1332 pg/mL, IQR: 561–3280). Patients who reached the combined endpoint had higher biomarkers of inflammation at baseline, including a median NLR (4.0 vs. 3.4), median MLR (0.5 vs. 0.4), median PIV (551 vs. 434), and median C-reactive protein (0.6 vs. 0.2 mg/dL, *p* ≤ 0.018 for all), compared to patients who did not meet the combined endpoint.

### 3.2. Imaging Parameters

Table 2 summarizes echocardiographic and CMR data at baseline. The median interval between baseline CMR and AVR was 21 days (IQR: 11–49). The patient cohort presented with preserved left (LVEF: 60%, IQR: 47–67) and right ventricular ejection fraction (RVEF: 54%, IQR: 45–61), alongside elevated median native two-chamber myocardial T1 times (1029 ms, IQR: 1007–1053) and ECV values (26.6%, IQR: 24.6–28.8) on CMR. LGE was present in 165 (46%) individuals. The inter-observer reliability of CMR-derived determinants of myocardial fibrosis demonstrated strong agreement in a subset of 35 patients (Supplementary Table S1).

### 3.3. Association of Inflammatory Indices with Clinical and Imaging Parameters

NLR, MLR, and PIV demonstrated statistically significant correlations with body mass index, NT-proBNP, and C-reactive protein levels, AV mean pressure gradient, systolic pulmonary artery pressure, and ECV at baseline. However, these correlations were generally weak (Supplementary Table S2). By linear regression analyses, only C-reactive protein emerged as an independent predictor for all three inflammatory indices. Additionally, body mass index was identified as an independent predictor for NLR, while AV mean pressure gradient independently predicted MLR and PIV. Detailed results of the linear regression analysis are presented in Supplementary Tables S3–S5.

### 3.4. Cardiovascular Outcomes

**Figure 2:**
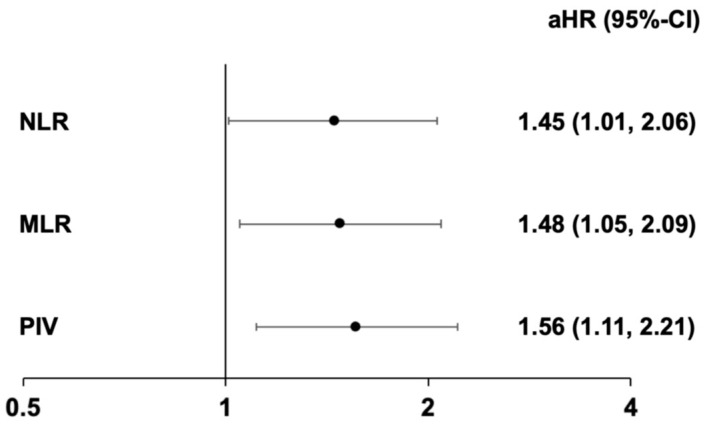
Cox regression analyses demonstrating the association between inflammatory indices and the combined endpoint (heart failure [HF] hospitalizations and death). After adjusting for clinical (EuroSCORE II), laboratory (baseline NT-proBNP and C-reactive protein levels), and imaging parameters (aortic valve mean pressure gradient, right ventricular ejection fraction, and extracellular volume), the above-the-upper-quartile neutrophil–lymphocyte ratio (NLR), monocyte–lymphocyte ratio (MLR), and pan-immune inflammation value (PIV) remained independent predictors of outcome. Abbreviations: aHR indicates adjusted hazard ratio; CI, confidence interval.

**Figure 3:**
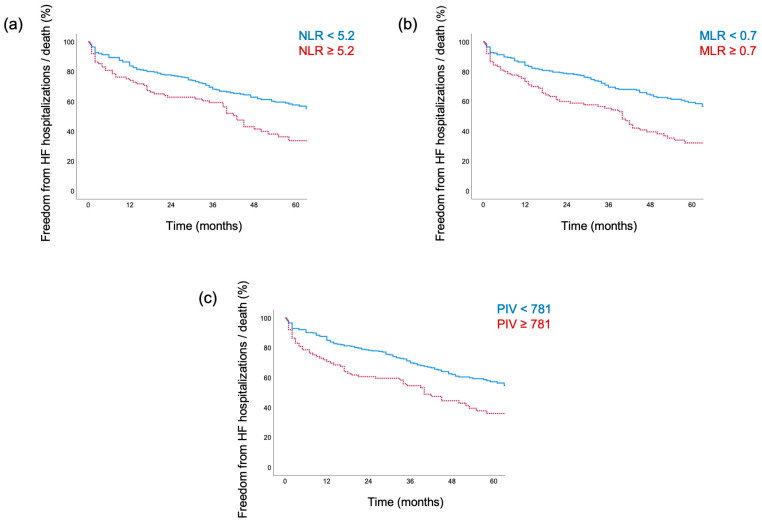
Kaplan–Meier estimators demonstrating differences in time to combined endpoint (heart failure [HF] hospitalizations and death) between the above-the-upper-quartile, (**a**) neutrophil–lymphocyte ratio (NLR, log-rank: *p* = 0.001), (**b**) monocyte–lymphocyte ratio (MLR, log-rank: *p* < 0.001), and (**c**) pan-immune inflammation value (PIV, log-rank: *p* < 0.001) at baseline.

A total of 162 events (141 deaths, 50 HF hospitalizations, and 29 instances of both) occurred over a median follow-up period of 40 months (IQR 21–60). The primary cause of death was cardiovascular (75%), followed by non-cardiovascular causes, including cancer progression (13%), infectious disease (9%), and various other causes (3%). In univariable Cox regression analyses, associations with the combined endpoint were demonstrated for all tested biomarkers of inflammation (above-the-upper-quartile): C-reactive protein (HR: 2.28, 95%-CI: 1.65–3.15), the NLR (HR: 1.72, 95%-CI: 1.24–2.40), MLR (HR: 1.98, 95%-CI: 1.44–2.73), and PIV (HR: 1.76, 95%-CI: 1.27–2.45). Subsequently, all inflammatory indices were tested after adjusting for clinical (EuroSCORE II), laboratory (baseline NT-proBNP and C-reactive protein levels), and imaging parameters (AV mean pressure gradient, RVEF, and ECV), which demonstrated significant associations at a univariable level. Variables already incorporated in the EuroSCORE II were excluded for further analysis. In multivariable Cox regression analyses, the above-the-upper-quartile NLR (aHR: 1.45, 95%-CI: 1.01–2.06), MLR (aHR: 1.48, 95%-CI: 1.05–2.09), and PIV (aHR: 1.56, 95%-CI: 1.11–2.21) remained significantly associated with the combined endpoint. Detailed results of the Cox regression analysis are presented in Table 3 and Figure 2. Kaplan–Meier curves illustrating the associations between inflammatory indices and the combined endpoint are shown in Figure 3. Additionally, a multivariable Cox regression analysis was conducted for continuous inflammatory indices, with detailed results provided in Supplementary Table S6.

Concerning the secondary endpoint of all-cause mortality, a significant association following multivariable adjustment was observed only for the above-the-upper-quartile MLR (aHR: 1.47, 95%-CI: 1.01–2.13). All results of the Cox regression analysis for the secondary endpoint are depicted in Supplementary Table S7.

After AVR, inflammatory biomarkers were available for 222 patients (62.4%) with a median follow-up of 12 months (IQR 5–30). The median NLR decreased from 3.5 to 3.4 (*p* = 0.019), and the PIV declined from 460 to 376 (*p* ≤ 0.001), both demonstrating significant reductions following AVR. In contrast, median C-reactive protein (0.3 mg/dL) and the MLR (0.5) remained unchanged (*p* ≥ 0.473 for both). Detailed follow-up data are shown in Supplementary Table S8.

## 4. Discussion

In this study, we demonstrated that (1) elevated baseline inflammatory indices are independently associated with HF hospitalizations and all-cause mortality in patients with severe AS, and (2) both the NLR and PIV significantly decrease following AVR. However, (3) no significant relationship was observed between systemic inflammatory biomarkers and myocardial fibrosis, as assessed by CMR.

While the prognostic value of inflammatory biomarkers has been investigated in other cardiac diseases [22], our study represents the first to directly compare the mid-term clinical outcomes of all three indices in AS patients. Previously, a post hoc analysis of the PARTNER trials demonstrated that a NLR above the upper quartile was significantly associated with mortality or rehospitalization at three years following AVR [23]. We were able to extend these findings by incorporating CMR imaging data, including a comprehensive assessment of myocardial fibrosis, which has previously been linked to inflammatory processes within the myocardium [24,25]. However, the extent to systemic inflammation, as indicated by the NLR, MLR, and PIV, translates into local myocardial alterations in patients with severe AS remains uncertain. Our findings demonstrate no association between these inflammatory indices, the presence of LGE, or elevated ECV on CMR. This aligns with preliminary data from Thompson et al. in HF patients, suggesting that the adverse prognosis linked to an elevated NLR may be driven by worsening of congestion and coronary artery disease. Notably, their study also found no significant relationship between the NLR and myocardial inflammation on CMR [26]. This discrepancy may stem from the specific inflammatory markers investigated, which may not directly reflect the intricate pathways involved in developing myocardial fibrosis.

At our research center, consistent with the findings of this study, we have previously shown that across the HF spectrum, various inflammatory indices correlate with disease severity and are associated with poor survival, comparable to cancer patients [6,27]. However, whether elevated inflammatory indices causally contribute to AS development and an increased risk of unfavorable outcomes following AVR or merely reflect underlying comorbidities associated with higher mortality remains debatable. Supporting a potential causal role, our study demonstrates that all three inflammatory indices retain prognostic significance even after adjustment for clinical, laboratory, and imaging parameters. Additionally, longitudinal data show a significant reduction in the NLR and PIV following AVR, suggesting a dynamic interplay between systemic inflammation and disease progression.

## 5. Clinical Implications and Future Directions

Differential blood counts, including the NLR, MLR, and PIV, are cost-effective and widely accessible inflammatory markers. Incorporating these indices into established pre-interventional risk assessment tools, such as the EuroSCORE II, may improve patient selection and risk stratification and facilitates timely referral to specialized centers prior to AVR. As anti-inflammatory therapies for HF patients continue to be explored, the potential role of inflammatory indices in guiding patient selection and monitoring disease progression warrants further investigation [15,28]. Moreover, advancements in AVR techniques and the reduction in procedural complications, such as significant paravalvular leak, may further modulate systemic inflammation and HF, subsequently affecting clinical outcomes in patients undergoing valvular treatment. Future research is warranted to integrate inflammatory biomarkers with advanced cardiovascular imaging to elucidate the complex interplay between chronic systemic inflammation, AS progression, and myocardial fibrosis. This approach may help to identify potential therapeutic targets and improve outcomes in AS patients undergoing AVR.

## 6. Limitations

All data were collected from a single center, possibly introducing a potential selection bias. However, the single-center setting ensures consistency in echocardiographic and CMR scanning conditions and post-processing workflows throughout the study period. The use of CMR imaging in clinical routine is complex and limited by cost- and time-consuming measurements. Consequently, not all patients undergoing AVR were eligible for participation, primarily due to the presence of implantable devices and limited scanner availability. Therefore, the possibility of selection bias cannot be entirely excluded. However, in a previous study based on the same cohort, we observed no significant differences between the CMR subgroup and the overall study population [29]. Although flow cytometry was not performed, it could be valuable in differentiating sub-populations of neutrophils, monocytes, and lymphocytes, which may have distinct roles in systemic inflammation. Additionally, the potential impact of SGLT2 inhibitors on inflammatory markers cannot be excluded, although their use was limited within this patient population. Although the event rate in our study population was high (45.5%), it remains within the range demonstrated in previous short- to mid-term follow-up studies, which reported rates between 28.2 and 58.4% [6,23,27].

## 7. Conclusions

Inflammatory indices are independently associated with HF hospitalizations and mid-term outcomes in AS patients undergoing AVR. However, no significant relationship was found between biomarkers of systemic inflammation and myocardial fibrosis on CMR.

## Figures and Tables

**Table 1:** Baseline characteristics of the patient cohort.

	All Patients (*n* = 356)	Combined Endpoint Met (*n* = 162)	Combined Endpoint Not Met (*n* = 194)	*p* Value
Clinical parameters				
Age (years)	80 (77–85)	82 (78–86)	79 (76–83)	<0.001
Male sex, *n* (%)	177 (50)	84 (52)	93 (48)	0.462
Body mass index (kg/m^2^)	27 (24–30)	26 (23–30)	27 (24–31)	0.034
EuroSCORE II (%)	4.1 (3.7–4.8)	4.4 (3.9–5.3)	3.9 (3.3–4.3)	<0.001
NYHA functional class ≥ III, *n* (%)	159 (45)	71 (44)	88 (45)	0.772
CCS class ≥ III, *n* (%)	35 (10)	18 (11)	17 (9)	0.459
Syncopes, *n* (%)	42 (12)	22 (14)	20 (10)	0.341
NT-proBNP (pg/mL)	1332 (561–3280)	2210 (912–5848)	923 (375–1862)	<0.001
Creatinine (mg/dL)	1.0 (0.8–1.3)	1.1 (0.9–1.6)	1.0 (0.8–1.2)	<0.001
eGFR (mL/min/1.73 m^2^)	64 (48–81)	56 (40–74)	71 (55–87)	<0.001
Comorbidities				
Coronary artery disease, *n* (%)	167 (47)	84 (52)	83 (43)	0.088
Myocardial infarction, *n* (%)	20 (6)	9 (6)	11 (6)	0.963
Percutaneous coronary intervention, *n* (%)	123 (35)	60 (37)	63 (33)	0.367
Coronary artery bypass graft, *n* (%)	35 (10)	22 (14)	13 (7)	0.030
Previous valve surgery, *n* (%)	28 (8)	14 (9)	14 (7)	0.619
Atrial fibrillation, *n* (%)	124 (35)	62 (38)	62 (32)	0.213
Arterial hypertension, *n* (%)	295 (83)	133 (82)	162 (84)	0.726
Diabetes mellitus type II, *n* (%)	102 (29)	48 (30)	54 (28)	0.709
Hyperlipidemia, *n* (%)	109 (31)	41 (25)	68 (35)	0.047
Previous stroke, *n* (%)	50 (14)	27 (17)	23 (12)	0.193
Cerebral artery disease, *n* (%)	66 (19)	32 (20)	34 (18)	0.590
Peripheral artery disease, *n* (%)	27 (8)	20 (12)	7 (4)	0.002
COPD, *n* (%)	42 (12)	30 (19)	12 (6)	<0.001
Concomitant medication				
Beta blockers, *n* (%)	221 (62)	102 (63)	119 (61)	0.753
ACE inhibitors, *n* (%)	120 (34)	52 (32)	68 (35)	0.557
Angiotensin receptor blockers, *n* (%)	136 (38)	61 (38)	75 (39)	0.846
ARNIs, *n* (%)	1 (<1)	1 (<1)	0 (0)	0.455
SGLT2 inhibitors, *n* (%)	18 (5)	3 (2)	15 (8)	0.014
Spironolactone, *n* (%)/daily dose (mg)	111 (31)/50 (25–50)	64 (40)/50 (50–50)	47 (24)/50 (25–50)	0.002
Loop diuretics, *n* (%)/daily dose (mg)	145 (41)/40 (30–60)	81 (50)/40 (40–60)	64 (33)/40 (20–40)	0.001
Thiazide diuretics, *n* (%)/daily dose (mg)	89 (25)/12.5 (12.5–16.3)	42 (26)/12.5 (12.5–21.3)	47 (24)/12.5 (12.5–12.5)	0.712
Oral anticoagulants, *n* (%)	149 (42)	77 (48)	72 (37)	0.047
Procedural data				
TAVR, *n* (%)	320 (90)	146 (90)	174 (90)	0.893
SAVR, *n* (%)	18 (5)	2 (1)	16 (8)	0.003
No valvular intervention, *n* (%)	18 (5)	14 (9)	4 (2)	0.006
Markers of inflammation				
Leukocytes (G/L)	7.1 (5.9–8.4)	6.9 (5.7–8.4)	7.3 (5.9–8.4)	0.213
Neutrophils (G/L)	4.9 (3.9–6.1)	4.9 (3.8–6.1)	5.0 (3.9–6.1)	0.503
Monocytes (G/L)	0.6 (0.5–0.8)	0.6 (0.5–0.8)	0.6 (0.5–0.7)	0.162
Lymphocytes (G/L)	1.3 (1.0–1.7)	1.1 (0.9–1.6)	1.4 (1.1–1.8)	<0.001
Thrombocytes (G/L)	210 (175–255)	213 (175–266)	209 (175–248)	0.436
C-reactive protein (mg/dL)	0.3 (0.1–1.1)	0.6 (0.2–1.7)	0.2 (0.1–0.6)	<0.001
NLR	3.7 (2.6–5.2)	4.0 (2.8–5.9)	3.4 (2.5–4.9)	0.004
MLR	0.5 (0.3–0.7)	0.5 (0.4–0.8)	0.4 (0.3–0.6)	<0.001
PIV	454 (276–781)	551 (267–950)	434 (277–671)	0.018

Values are given as median and interquartile range (IQR) or n (%). Abbreviations: NYHA indicates New York Heart Association; CCS, Canadian Cardiovascular Society; NT-proBNP, N-terminal prohormone of brain natriuretic peptide; eGFR, estimated glomerular filtration rate; COPD, chronic obstructive pulmonary disease; ACE, angiotensin converting enzyme; ARNI, angiotensin receptor-neprilysin inhibitor; SGLT2, sodium-glucose cotransporter-2; TAVR, transcatheter aortic valve replacement; SAVR, surgical aortic valve repair; NLR, neutrophil–lymphocyte ratio; MLR, monocyte–lymphocyte ratio; PIV, pan-immune inflammation value.

**Table 2:** Imaging parameters of the patient cohort.

	All Patients (*n* = 356)	Combined Endpoint Met (*n* = 162)	Combined Endpoint Not Met (*n* = 194)	*p* Value
Echocardiography				
LV end-diastolic diameter (mm)	44 (39–48)	44 (40–48)	43 (39–48)	0.223
RV end-diastolic diameter (mm)	32 (29–36)	33 (30–38)	32 (27–35)	<0.001
Interventricular septum (mm)	15 (13–17)	14 (13–17)	15 (13–16)	0.996
LV ejection fraction (%)	55 (52–63)	55 (45–61)	55 (55–65)	0.019
AV mean pressure gradient (mmHg)	45 (36–54)	43 (33–52)	47 (39–56)	0.008
AV peak pressure gradient (mmHg)	73 (59–86)	70 (53–84)	73 (64–88)	0.006
AV Vmax (m/s)	4.3 (3.9–4.7)	4.2 (3.7–4.6)	4.3 (4.0–4.7)	0.013
AV area index (cm^2^/m^2^)	0.7 (0.6–0.8)	0.7 (0.6–0.8)	0.7 (0.6–0.8)	0.811
Systolic PAP (mmHg)	48 (37–61)	48 (40–61)	46 (35–61)	0.152
TAPSE (mm)	21 (18–24)	20 (17–24)	22 (19–24)	0.123
RV FAC (%)	46 (39–55)	44 (37–53)	47 (44–55)	0.028
Mitral regurgitation ≥ moderate, *n* (%)	74 (21)	46 (28)	28 (14)	0.001
Tricuspid regurgitation ≥ moderate, *n* (%)	72 (20)	46 (28)	26 (13)	<0.001
CMR				
LV end-diastolic volume (mL)	145 (113–185)	155 (118–192)	139 (110–174)	0.054
LV end-systolic volume (mL)	57 (38–95)	66 (41–106)	52 (36–84)	0.005
LV cardiac index (L/min/m^2^)	3.0 (2.5–3.5)	2.8 (2.4–3.5)	3.1 (2.6–3.5)	0.029
LV ejection fraction (%)	60 (47–67)	57 (37–67)	62 (51–68)	<0.001
LV global longitudinal strain (-%)	13 (10–16)	13 (9–15)	14 (11–17)	0.002
LV mass index (g/m^2^)	78 (63–94)	80 (65–96)	75 (62–91)	0.161
Interventricular septum (mm)	13 (11–15)	13 (11–15)	13 (11–15)	0.423
RV end-diastolic volume (mL)	136 (112–175)	147 (114–194)	132 (110–164)	0.010
RV end-systolic volume (mL)	64 (48–88)	71 (53–104)	60 (45–79)	<0.001
RV cardiac index (L/min/m^2^)	2.7 (2.3–3.2)	2.7 (2.3–3.3)	2.7 (2.3–3.2)	0.738
RV ejection fraction (%)	54 (45–61)	51 (40–60)	55 (49–61)	<0.001
Presence of LGE, *n* (%)	165 (46)	79 (51)	86 (44)	0.240
Native 2ch myocardial T1 times (ms)	1029 (1007–1053)	1039 (1016–1073)	1019 (1004–1044)	<0.001
ECV (%)	26.6 (24.6–28.8)	28.0 (25.7–31.0)	25.8 (24.0–27.5)	<0.001

Values are given as median and interquartile range (IQR) or n (%). Abbreviations: LV indicates left ventricular; RV, right ventricular; AV, aortic valve; Vmax, peak jet velocity; PAP, pulmonary artery pressure; TAPSE, tricuspid annular plane systolic excursion; FAC, fractional area change; CMR, cardiovascular magnetic resonance; LGE, late gadolinium enhancement; 2ch, two-chamber; ECV, extracellular volume.

**Table 3:** Cox regression analyses for the combined endpoint of all-cause mortality and heart failure hospitalization. Multivariable analysis was adjusted for all clinical (EuroSCORE II, baseline NT-proBNP and C-reactive protein levels) and imaging parameters (AV mean pressure gradient, RV ejection fraction, and ECV), with a significant influence at a univariable level, excluding variables already incorporated in the EuroSCORE II.

	Univariable Analysis			Multivariable Analysis		
	HR	95% CI	*p* Value	aHR	95% CI	*p* Value
Clinical parameters						
Age	1.05	1.02–1.08	<0.001			
Male sex	1.20	0.88–1.63	0.247			
EuroSCORE II ≥ 4%	2.25	1.61–3.17	<0.001	1.50	1.02–2.21	0.040
NT-proBNP (logarithmized)	2.60	1.96–3.44	<0.001	1.55	1.09–2.19	0.014
eGFR	0.98	0.98–0.99	<0.001			
Markers of inflammation						
C-reactive protein (above Q3)	2.28	1.65–3.15	<0.001	1.32	0.90–1.92	0.155
Echocardiography						
LV ejection fraction	0.98	0.97–0.99	<0.001			
AV mean pressure gradient	0.99	0.98–1.00	0.003	1.00	1.00–1.00	0.642
Systolic PAP	1.01	1.00–1.02	0.043			
TAPSE	0.97	0.93–1.00	0.074			
RV FAC	0.14	0.02–0.79	0.026			
CMR						
LV ejection fraction	0.98	0.97–0.99	<0.001			
LV global longitudinal strain	0.93	0.89–0.97	<0.001			
RV ejection fraction < 45%	2.13	1.53–2.96	<0.001	1.28	0.86–1.91	0.229
Presence of LGE	1.23	0.90–1.68	0.202			
Native 2ch myocardial T1 times	1.01	1.01–1.01	<0.001			
ECV (median)	3.05	2.15–4.33	<0.001	2.33	1.59–3.42	<0.001
Inflammatory indices						
NLR (above Q3)	1.72	1.24–2.40	0.001	1.45	1.01–2.06	0.042
MLR (above Q3)	1.98	1.44–2.73	<0.001	1.48	1.05–2.09	0.026
PIV (above Q3)	1.76	1.27–2.45	<0.001	1.56	1.11–2.21	0.011

Abbreviations: NT-proBNP indicates N-terminal prohormone of brain natriuretic peptide; AV, aortic valve; HR indicates hazard ratio; RV, right ventricular; ECV, extracellular volume; CI, confidence interval; aHR, adjusted HR; eGFR, estimated glomerular filtration rate; Q3, upper/third quartile; LV, left ventricular; PAP, pulmonary artery pressure; TAPSE, tricuspid annular plane systolic excursion; FAC, fractional area change; CMR, cardiovascular magnetic resonance; LGE, late gadolinium enhancement; 2ch, two-chamber; NLR, neutrophil–lymphocyte ratio; MLR, monocyte–lymphocyte ratio; PIV, pan-immune inflammation value.

## Data Availability

The data presented in this study are available on request from the corresponding author.

## Supplementary Materials

The following supporting information can be downloaded at https://www.mdpi.com/article/10.3390/jcm14072512/s1, Table S1: Inter-observer reliability of CMR-derived determinants of myocardial fibrosis in a subset of 35 patients; Table S2: Correlation analyses of leukocyte indices with clinical and imaging parameters at baseline; Table S3: Linear regression analyses demonstrating the association between clinical and imaging parameters and the neutrophil-lymphocyte ratio at baseline; Table S4: Linear regression analyses demonstrating the association between clinical and imaging parameters and the monocyte-lymphocyte ratio at baseline; Table S5: Linear regression analyses demonstrating the association between clinical and imaging parameters and the pan-immune inflammation value at baseline; Table S6: Cox regression analyses for the combined endpoint of all-cause mortality and heart failure hospitalization; Table S7: Cox regression analyses for the secondary endpoint of all-cause mortality; Table S8: Comparisons between biomarkers of inflammation at baseline and after AVR.

## Abbreviations

AS	Aortic stenosis
AVR	Aortic valve replacement
CMR	Cardiovascular magnetic resonance
ECV	Extracellular volume
LGE	Late gadolinium enhancement
MLR	Monocyte-to-lymphocyte ratio
NLR	Neutrophil-to-lymphocyte ratio
PIV	Pan-immune inflammation value
