# Supplementary material for: Biomarkers of Inflammation and Association with Cardiovascular Magnetic Resonance Imaging for Risk Stratification and Outcome in Patients with Severe Aortic Stenosis
Source: J Clin Med. 2025 Apr 7;14(7):2512. doi: 10.3390/jcm14072512 (PMC11990065; doi:10.3390/jcm14072512)
Supplement: Supplementary file 1 [file jcm-14-02512-s001.zip › jcm-3480181-supplementary.pdf]

Supplementary Materials

Supplemental Table S1. Inter-observer reliability of CMR-derived determinants of myocardial fibrosis in a subset of 35 patients.

	**ICC**	**95% CI**	***P* value**
**CMR**			
Presence of LGE	0.971	0.943-0.986	**<0.001**
Native 2ch myocardial T1 times	0.977	0.949-0.990	**<0.001**
ECV	0.966	0.958-0.972	**<0.001**

Abbreviations: CMR indicates cardiovascular magnetic resonance; ICC, intraclass correlation coefficient; CI, confidence interval; LGE, late gadolinium enhancement; 2ch, two-chamber; ECV, extracellular volume.

Supplemental Table S2. Correlation analyses of leukocyte indices with clinical and imaging parameters at baseline.

	NLR		MLR		PIV	
	**Coeff.**	***P* value**	**Coeff.**	***P* value**	**Coeff.**	***P* value**
**Clinical parameters**						
Age (years)	0.056	0.291	0.065	0.224	0.031	0.563
Male sex, n (%)	0.053	0.318	0.142	**0.007**	-0.047	0.375
Body mass index (kg/m^2)	-0.189	**<0.001**	-0.147	**0.006**	-0.138	**0.009**
EuroSCORE II (%)	0.104	0.051	0.148	**0.005**	0.065	0.220
NT-proBNP (pg/mL)	0.284	**<0.001**	0.246	**<0.001**	0.145	**0.007**
eGFR (mL/min/1.73m^2)	-0.099	0.061	-0.113	**0.033**	-0.026	0.628
**Markers of inflammation**						
Leukocytes (G/L)	0.230	**<0.001**	-0.020	0.709	0.500	**<0.001**
Neutrophils (G/L)	0.527	**<0.001**	0.145	**0.006**	0.651	**<0.001**
Monocytes (G/L)	0.152	**0.004**	0.511	**<0.001**	0.605	**<0.001**
Lymphocytes (G/L)	-0.765	**<0.001**	-0.757	**<0.001**	-0.411	**<0.001**
Thrombocytes (G/L)	0.086	0.107	0.012	0.829	0.505	**<0.001**
C-reactive protein (mg/dL)	0.313	**<0.001**	0.322	**<0.001**	0.350	**<0.001**
**Echocardiography**						
LV ejection fraction (%)	-0.119	**0.025**	-0.132	**0.012**	-0.064	0.227

AV mean pressure gradient (mmHg)	-0.196	**<0.001**	-0.184	**<0.001**	-0.212	**<0.001**
Systolic PAP (mmHg)	0.161	**0.008**	0.167	**0.006**	0.142	**0.020**
TAPSE (mm)	-0.110	0.150	-0.121	0.112	-0.103	0.175
RV FAC (%)	-0.172	**0.027**	-0.204	**0.009**	-0.052	0.506
**CMR**						
LV end-diastolic volume (mL)	0.084	0.120	0.062	0.247	-0.093	**0.084**
LV end-systolic volume (mL)	0.140	**0.009**	0.129	**0.016**	-0.012	0.830
LV ejection fraction (%)	-0.190	**<0.001**	-0.187	**<0.001**	-0.083	0.124
LV global longitudinal strain (-%)	-0.091	0.097	-0.097	0.075	-0.046	0.404
Interventricular septum (mm)	-0.106	**0.048**	-0.079	0.144	-0.108	**0.045**
RV end-diastolic volume (mL)	0.076	0.156	0.119	**0.026**	-0.026	0.630
RV end-systolic volume (mL)	0.121	**0.025**	0.165	**0.002**	0.030	0.573
RV ejection fraction (%)	-0.143	**0.008**	-0.167	**0.002**	-0.088	0.103
Presence of LGE, n (%)	0.029	0.586	0.011	0.845	-0.035	0.519
Native 2ch myocardial T1 times (ms)	0.088	0.100	0.095	0.076	0.060	0.263
ECV (%)	0.219	**<0.001**	0.219	**<0.001**	0.142	**0.009**

Abbreviations: NLR indicates neutrophil-lymphocyte ratio; MLR, monocyte-lymphocyte ratio; PIV, pan-immune inflammation value; Coeff., Spearman correlation coefficient; NT-proBNP, N-terminal prohormone of brain natriuretic peptide; eGFR, estimated glomerular filtration rate; LV, left ventricular; AV, aortic valve; PAP, pulmonary artery pressure; TAPSE, tricuspid annular plane systolic excursion; RV, right ventricular; FAC, fractional area change; CMR, cardiovascular magnetic resonance; LGE, late gadolinium enhancement; 2ch, two-chamber; ECV, extracellular volume.

Supplemental Table S3. Linear regression analyses demonstrating the association between clinical and imaging parameters and the neutrophil-lymphocyte ratio at baseline. Multivariable analysis was adjusted for all clinical (body mass index, baseline NT-proBNP and C-reactive protein levels), and imaging parameters (AV mean pressure gradient, systolic PAP, LV ejection fraction, RV ejection fraction, and ECV) with a significant influence at an univariable level.

**NLR**	**Univariable analysis**			**Multivariable analysis**		
	**Coeff.**	**95% CI**	***P* value**	**Adj. Coeff.**	**95% CI**	***P* value**
**Clinical parameters**						
Age	0.037	-0.006-0.080	0.095			
Male sex	0.025	-0.529-0.579	0.980			
Body mass index	-0.079	-0.139-(-)0.020	**0.009**	-0.080	-0.151-(-)0.010	**0.026**
EuroSCORE II	0.033	-0.052-0.079	0.680			
NT-proBNP (logarithmized)	1.15	0.69-1.62	**<0.001**	0.357	-0.376-1.090	0.338
eGFR	-0.009	-0.020-0.002	0.097			
**Markers of inflammation**						
C-reactive protein	0.620	0.419-0.821	**<0.001**	0.543	0.264-0.822	**<0.001**
**Echocardiography**						
LV ejection fraction	-0.027	-0.049-(-)0.005	**0.017**			
AV mean pressure gradient	-0.028	-0.044-(-)0.011	**<0.001**	-0.018	-0.037-0.001	0.067
Systolic PAP	0.025	0.005-0.044	**0.013**	0.010	-0.012-0.032	0.353

**CMR**						
LV ejection fraction	-0.032	-0.050-(-)0.015	**<0.001**	0.002	-0.030-0.034	0.906
LV global longitudinal strain	-0.055	-0.121-0.010	0.095			
RV ejection fraction	-0.048	-0.071-(-)0.025	**<0.001**	-0.012	-0.052-0.028	0.552
Presence of LGE	0.221	-0.333-0.775	0.433			
Native 2ch myocardial T1 times	0.008	0.001-0.015	**0.022**			
ECV	0.138	0.077-0.199	**<0.001**	0.041	-0.039-0.122	0.313

Abbreviations: NT-proBNP indicates N-terminal prohormone of brain natriuretic peptide; AV, aortic valve; PAP, pulmonary artery pressure; LV, left ventricular; RV, right ventricular; ECV, extracellular volume; NLR, neutrophil-lymphocyte ratio; Coeff. indicates coefficient; CI, confidence interval; Adj., adjusted; eGFR, estimated glomerular filtration rate; CMR, cardiovascular magnetic resonance; LGE, late gadolinium enhancement; 2ch, two-chamber.

Supplemental Table S4. Linear regression analyses demonstrating the association between clinical and imaging parameters and the monocyte-lymphocyte ratio at baseline. Multivariable analysis was adjusted for all clinical (male sex, baseline NT-proBNP and C-reactive protein levels), and imaging parameters (AV mean pressure gradient, systolic PAP, LV ejection fraction, RV ejection fraction, and ECV) with a significant influence at an univariable level.

**MLR**	**Univariable analysis**			**Multivariable analysis**		
	**Coeff.**	**95% CI**		**Coeff.**	**95% CI**	
**Clinical parameters**						
Age	0.003	-0.002-0.008	0.272			
Male sex	0.072	0.004-0.140	**0.037**	0.052	-0.027-0.131	0.192
Body mass index	-0.006	-0.014-0.001	0.105			
EuroSCORE II	0.005	-0.003-0.013	0.198			
NT-proBNP (logarithmized)	0.112	0.054-0.170	**<0.001**	0.050	-0.038-0.139	0.263
eGFR	-0.001	-0.002-0.000	0.105			
**Markers of inflammation**						
C-reactive protein	0.062	0.037-0.088	**<0.001**	0.046	0.012-0.079	**0.007**
**Echocardiography**						
LV ejection fraction	-0.003	-0.005-0.000	**0.049**			
AV mean pressure gradient	-0.004	-0.006-(-)0.002	**<0.001**	-0.002	-0.005-0.000	**0.042**
Systolic PAP	0.004	0.002-0.007	**<0.001**	0.002	0.000-0.005	0.062

**CMR**						
LV ejection fraction	-0.003	-0.005-(-)0.001	**0.003**	0.001	-0.003-0.005	0.656
LV global longitudinal strain	-0.007	-0.015-0.001	0.098			
RV ejection fraction	-0.006	-0.009-(-)0.003	**<0.001**	-0.001	-0.006-0.004	0.702
Presence of LGE	0.001	-0.065-0.067	0.986			
Native 2ch myocardial T1 times	0.001	0.000-0.002	**0.012**			
ECV	0.019	0.011-0.027	**<0.001**	0.008	-0.001-0.018	0.090

Abbreviations: NT-proBNP indicates N-terminal prohormone of brain natriuretic peptide; AV, aortic valve; PAP, pulmonary artery pressure; LV, left ventricular; RV, right ventricular; ECV, extracellular volume; MLR, monocyte-lymphocyte ratio; Coeff. indicates coefficient; CI, confidence interval; Adj., adjusted; eGFR, estimated glomerular filtration rate; CMR, cardiovascular magnetic resonance; LGE, late gadolinium enhancement; 2ch, two-chamber.

Supplemental Table S5. Linear regression analyses demonstrating the association between clinical and imaging parameters and the pan-immune inflammation value at baseline. Multivariable analysis was adjusted for all clinical (baseline NT-proBNP and C-reactive protein levels), and imaging parameters (AV mean pressure gradient, systolic PAP, LV ejection fraction, RV ejection fraction, and ECV) with a significant influence at an univariable level.

**PIV**	**Univariable analysis**			**Multivariable analysis**		
	**Coeff.**	**95% CI**	***P* value**	**Adj. Coeff.**	**95% CI**	***P* value**
**Clinical parameters**						
Age	3.27	-5.45-11.99	0.461			
Male sex	-56	-168-55	0.321			
Body mass index	-8.59	-20.83-3.66	0.169			
EuroSCORE II	6.09	-7.15-19.33	0.366			
NT-proBNP (logarithmized)	174	79-270	**<0.001**	91	-71-253	0.270
eGFR	-1.47	-3.69-0.75	0.194			
**Markers of inflammation**						
C-reactive protein	125	84-165	**<0.001**	148	94-203	**<0.001**
**Echocardiography**						
LV ejection fraction	-3.78	-8.22-0.70	0.098			
AV mean pressure gradient	-6.41	-9.69-(-)3.13	**<0.001**	-5.21	-9.45-(-)0.96	**0.017**
Systolic PAP	4.59	0.39-8.79	**0.032**	1.72	-3.10-6.53	0.483

**CMR**						
LV ejection fraction	-4.53	-8.13-(-)0.93	**0.014**	3.02	-4.03-10.07	0.400
LV global longitudinal strain	-13	-27-(-)0	**0.045**			
RV ejection fraction	-7.88	-12.57-(-)3.19	**0.001**	-4.06	-12.71-4.59	0.356
Presence of LGE	15	-99-129	0.790			
Native 2ch myocardial T1 times	1.09	-0.34-2.54	**0.022**			
ECV	20	7-32	**0.003**	2.67	-15.14-20.48	0.768

Abbreviations: NT-proBNP indicates N-terminal prohormone of brain natriuretic peptide; AV, aortic valve; PAP, pulmonary artery pressure; LV, left ventricular; RV, right ventricular; ECV, extracellular volume; PIV, pan-immune inflammation value; Coeff. indicates coefficient; CI, confidence interval; Adj., adjusted; eGFR, estimated glomerular filtration rate; CMR, cardiovascular magnetic resonance; LGE, late gadolinium enhancement; 2ch, two-chamber.

Supplemental Table S6. Cox regression analyses for the combined endpoint of all-cause mortality and heart failure hospitalization. Multivariable analysis was adjusted for EuroSCORE II and NT-proBNP levels (logarithmized).

	**Univariable analysis**			**Multivariable analysis**		
	**HR**	**95% CI**	***P* value**	**aHR**	**95% CI**	***P* value**
**Continuous variables**						
**Inflammatory indices**						
NLR	1.11	1.06-1.17	**<0.001**	1.07	1.02-1.13	**0.011**
MLR	2.38	1.63-3.46	**<0.001**	1.78	1.18-2.67	**<0.001**
PIV	1.00	1.00-1.00	**<0.001**	1.00	1.00-1.00	**<0.001**

Abbreviations: NT-proBNP indicates N-terminal prohormone of brain natriuretic peptide; HR indicates hazard ratio; CI, confidence interval; aHR, adjusted HR; NLR, neutrophil-lymphocyte ratio; MLR, monocyte-lymphocyte ratio; PIV, pan-immune inflammation value.

Supplemental Table S7. Cox regression analyses for the secondary endpoint of all-cause mortality. Multivariable analysis was adjusted for all clinical (EuroSCORE II, baseline NT-proBNP and C-reactive protein levels), and imaging parameters (AV mean pressure gradient, RV ejection fraction, and ECV) with a significant influence at an univariable level, excluding variables already incorporated in the EuroSCORE II.

	**Univariable analysis**			**Multivariable analysis**		
	**HR**	**95% CI**	***P* value**	**aHR**	**95% CI**	***P* value**
**Clinical parameters**						
Age	1.05	1.02-1.08	**<0.001**			
Male sex	1.36	0.98-1.90	0.067			
EuroSCORE II ≥ 4%	2.47	1.70-3.58	**<0.001**	1.72	1.12-2.64	**0.013**
NT-proBNP (logarithmized)	2.80	2.08-3.77	**<0.001**	1.72	1.18-2.52	**0.005**
eGFR	0.98	0.98-0.99	**<0.001**			
**Markers of inflammation**						
C-reactive protein (above Q3)	2.28	1.62-3.22	**<0.001**	1.33	0.88-2.00	0.176
**Echocardiography**						
LV ejection fraction	0.97	0.96-0.99	**<0.001**			
AV mean pressure gradient	0.99	0.98-1.00	**0.018**	1.00	0.99-1.01	0.883
Systolic PAP	1.01	1.00-1.02	0.134			

TAPSE	0.97	0.93-1.01	0.115			
RV FAC	0.23	0.04-1.47	0.112			
**CMR**						
LV ejection fraction	0.98	0.97-0.99	**<0.001**			
LV global longitudinal strain	0.92	0.89-0.96	**<0.001**			
RV ejection fraction <45%	1.98	1.39-2.83	**<0.001**	1.16	0.75-1.78	0.515
Native 2ch myocardial T1 times	1.01	1.00-1.01	**<0.001**			
ECV (median)	2.81	1.93-4.08	**<0.001**	2.05	1.36-3.10	**<0.001**
**Leukocyte indices**						
NLR (above Q3)	1.76	1.24-2.51	**0.002**	1.39	0.94-2.05	0.101
MLR (above Q3)	2.02	1.44-2.84	**<0.001**	1.47	1.01-2.13	**0.045**
PIV (above Q3)	1.61	1.13-2.29	**0.009**	1.29	0.87-1.89	0.202

Abbreviations: NT-proBNP indicates N-terminal prohormone of brain natriuretic peptide; AV, aortic valve; HR indicates hazard ratio; RV, right ventricular; ECV, extracellular volume; CI, confidence interval; aHR, adjusted HR; eGFR, estimated glomerular filtration rate; Q3, upper/third quartile; LV, left ventricular; PAP, pulmonary artery pressure; TAPSE, tricuspid annular plane systolic excursion; FAC, fractional area change; CMR, cardiovascular magnetic resonance; 2ch, two-chamber; NLR, neutrophil-lymphocyte ratio; MLR, monocyte-lymphocyte ratio; PIV, pan-immune inflammation value.

Supplemental Table S8. Comparisons between biomarkers of inflammation at baseline and after AVR.

	**Baseline (n=222)**	**Follow-up (n=222)**	***P* value**
C-reactive protein (mg/dL)	0.3 (0.1-1.1)	0.3 (0.1-0.8)	0.519
NLR	3.5 (2.5-5.2)	3.4 (2.6-5.0)	**0.019**
MLR	0.5 (0.3-0.7)	0.5 (0.4-0.6)	0.473
PIV	460 (264-836)	376 (231-584)	**<0.001**

Values are given as median and interquartile range (IQR). Abbreviations: AVR indicates aortic valve replacement; NLR, neutrophil-lymphocyte ratio; MLR, monocyte-lymphocyte ratio; PIV, pan-immune inflammation value.
